# Does the ethnicity distribution of research participants reflect the eligible population? Survey of participants recruited through a UK mental health Trust

**DOI:** 10.1136/bmjopen-2024-093269

**Published:** 2025-03-19

**Authors:** Aikaterini Dima, Juliana Onwumere, Amanda Brown, Tanya Shlovogt, Silian Martinez, Maria Antonietta Nettis, Kia-Chong Chua, Matthew Hotopf, Fiona Gaughran

**Affiliations:** 1 South London and Maudsley NHS Foundation Trust, London, UK; 2 King’s College London Institute of Psychiatry Psychology and Neuroscience, London, UK; 3 Department of Psychology, King’s College London Institute of Psychiatry Psychology and Neuroscience, London, UK; 4 NIHR Maudsley Biomedical Research Centre, London, UK; 5 Department of Psychosis Studies, King’s College London Institute of Psychiatry Psychology and Neuroscience, London, UK; 6 Department of Psychological Medicine, King’s College London Institute of Psychiatry Psychology and Neuroscience, London, UK; 7 National Psychosis Service, South London and Maudsley NHS Foundation Trust, London, UK

**Keywords:** MENTAL HEALTH, Health Services, PSYCHIATRY

## Abstract

**Abstract:**

**Objectives:**

To compare the ethnicity distribution of research participants recruited through a UK mental health Trust with that of the population receiving clinical care in that Trust and the wider local population.

**Design:**

Survey of the ethnicity breakdown of participants in eligible studies, compared with Census data for the Trust catchment area and the Trust patient metrics.

**Setting:**

A London NHS mental health Trust.

**Participants:**

The survey was sent to principal investigators of studies opened and completed in the Trust between 2012 and 2022, that had recruited 90 or more participants. Data from 22 of 28 eligible studies were collected, yielding a sample of 3279 research participants.

**Results:**

Results indicated high alignment between research participant ethnicity and Trust patient population across five main ethnicity categories (Asian, Black, Mixed, White, Other). For example, people who identified as ‘any Black ethnic group’ comprised 24.5% of the Census population, 23.8% of the Trust clinical population and 25.4% of the research participant population. The study also identified areas for improvement, including in the recording of ethnicity and in consistency in terms and definitions used.

**Conclusions:**

Our findings indicate good levels of representation in relation to participant ethnicity in larger-scale research studies recruited through the Trust. Our work highlights the need for ongoing efforts to ensure representativeness in mental health research and for consistent and comprehensive reporting practices.

**STRENGTHS AND LIMITATIONS OF THIS STUDY**

- This survey is of research participants in a large urban mental healthcare provider which provides secondary care services to a defined, highly diverse geographical area, alongside some national services, thus allowing comparison of the ethnic representativeness of research participants with that of the Trust clinical population and local Census data.
- Studies included were large (90+participants) and were conducted over a 10-year period.
- Ethnicity is recorded in different ways across studies and settings.
- Data are presented in descriptive format only as statistical analyses across the different data sources would not be valid.

## Introduction

 Clinical research informs healthcare practice, access and provision, with the goal of improving outcomes for all healthcare users. Therefore, it has been recognised that inclusion in research should reflect the relevant population diversity in demographic, social and cultural backgrounds.^1^,^4^ Despite this, the limited strategies to increase diversity of recruitment^5^ have not yet translated to increased participation of minoritised ethnic groups in clinical research.^6^,^8^ This under-representation risks systematic ethnic inequalities in clinical care,^3 9^ creating a potential gap between available research findings and their applicability to multiethnic populations, especially in relation to personalised clinical care.

This gap has also been reported in mental health research. A 2022 quantification^10^ examining the demographics of participants in psychiatric research published in the American Journal of Psychiatry over a span of 2 years found that people categorised as from Asian, Black and minoritised ethnic groups were under-represented in research. Across 125 studies included, only 43% presented information on participant ethnicity, and in those, 94.6% were described as white or Caucasian. This figure dropped to 74.1% when only non-genetic studies were examined, with 10.6% described as ‘other’ or ‘nonwhite’, 7.6% as African American, Black or African and 5.3% as Hispanic or Latino. Such metrics risk diminishing the confidence of people from racially minoritised ethnic groups that the knowledge derived from research is relevant to their health needs. This is especially relevant in light of the 2022 UK National Health Service (NHS) Race and Health Observatory report,^11^ where barriers and disparities in accessing evidence-based mental health services for people from minoritised groups are highlighted.

Minoritised ethnic groups represent approximately 18% of the population of England and Wales^12^ and 24.5% of the USA population.^13^ While the National Institute for Health and Care Research in England has taken steps to promote inclusive research recruitment,^14^ we aimed to establish historical research representativeness in a large urban NHS mental health Trust over a 10-year period.

The main objective of our study was thus to examine whether research participation in a UK mental health setting reflects the population served in terms of ethnicity.

## Methods

### Setting

The South London and Maudsley NHS Foundation Trust (the Trust) is one of Europe’s largest secondary mental healthcare providers. It provides near-complete mental healthcare to a geographically distinct and ethnically diverse catchment area of roughly 1.36 million people in four South-East London boroughs, Croydon, Lambeth, Lewisham and Southwark, addiction services to three other Greater London boroughs, as well as selected national services. To assess the representativeness of research recruitment among the Trust, we compared the demographics of participants in ‘large’ mental health research studies conducted through the Trust, alongside the population recorded as receiving care within the Trust and Census data on the four London boroughs served by the Trust.

#### Research studies

##### Study identification

Studies were identified using the Trust’s clinical research management system, EDGE. EDGE is used across the NHS and enables users to track studies from beginning to end, as well as recording participant numbers.^15^ EDGE did not collect demographic data on research participants during the time frame examined.

Inclusion/eligibility criteria were:

- ‘Large’ studies, recorded on EDGE as having recruited at least 90 participants through the Trust. (Studies could be multicentre but only those recruiting >90 participants through the Trust were included). A threshold of 90 participants was taken for both pragmatic and information governance reasons.
- The study started after 1 April 2012 and concluded on or before 1 April 2022.

Studies preferentially recruited participants from a defined ethnic group or groups that were excluded. The full search strategy is provided in online supplemental material 1.

##### Data collection

Between April 2022 and February 2024, we contacted principal investigators (PIs) of eligible studies by email, who were asked to submit group demographic data of participants recruited through the Trust for the studies. Where needed, follow-up attempts were made to a maximum of five.

### Patient population

Within the mental health Trust, population-level patient data was gathered in December 2023 from the Trust online data dashboard, which is updated monthly.

### Local population

Local population data on the four London boroughs served by the Trust was taken from published Census 2021 data.^12^

### Data presentation and analysis

Data are presented descriptively as numbers and percentages. Where n<5, numbers were not presented to preserve anonymity. We opted for descriptive statistics rather than inferential due to the different sources of data and the category limitations. CIs were not used as the different source populations meant they would not be directly comparable.

Given that ethnicity was recorded in a different way for each research study, we combined groups as presented in online supplemental material 2, following the Census groupings (see online supplemental material 3).

### Missing data

Census data was imputed by the Census team and missing data ranged from 2.0 to 2.4%.^12^ Ethnicity data was unavailable for 13.8% of Trust patients and for 4.7% of research participants. We omitted missing data in the table displaying these groups together to allow for easier visual comparison.

## Results

We identified 28 eligible studies with a total of 4856 participants (range 94–555). All but eight were multicentre. 18 studies were in Adult Mental Health, two studies were in Addictions, five in Children and Adolescent Mental Health and three studies were jointly between Child and Adolescent Mental Health and Adult Mental Health. Three PIs were uncontactable or did not provide data for their studies (total n=376), of which two were interventional and one observational. Three further studies (total n=366, two interventional, one observational) advised that ethnicity data had not been collected. We received demographic data on 22 studies with a total of 3279 participants, half of which were interventional, half observational.

### Participants’ ethnicity background

Of the research participants, classified in the five main ethnic groups from the Census (see online supplemental material 3) including the 4.7% of participants with missing data, 52.1% were of any White background, 24.2% of any Black background and 5.9% of any Asian background. The ethnicity breakdown of the recruited participants as categorised by the research teams is provided in online supplemental material 4.

The equivalent figures from the Trust’s overall patient population were, including the higher figure of 13.8% with missing data, 45.0% of any White background, 20.6% of any Black background and 5.2% of any Asian background.

According to the Census, in the four South East London boroughs served by the Trust, 48.6% of residents identify as having minority ethnic status with a further 14.4% identifying with White ethnic minority status.

Table 1 shows, for ease of comparability, an overview of the ethnicity of the Trust research participants and the Trust service user population as a percentage of the available data, alongside the 2021 Census figures for the relevant London boroughs.

**Table 1:** High-level ethnicity across South East London Census data, Trust clinical population and Trust research participants

Ethnicity	South East London Census data, N (%)*	Trust patient population data, N (%)†	Research participants’ data, N (%)‡
Any Asian or Asian British background	149 005 (11.3)	2774 (6.1)	193 (6.2)
Any Black or Black British background	322 358 (24.5)	10 922 (23.8)	793 (25.4)
Any Mixed background	101 779 (7.7)	5103 (11.1)	241 (7.7)
Any other ethnic groups	66 693 (5.1)	3147 (6.9)	191 (6.1)
Any White or White British background	676 732 (51.4)	23 899 (52.1)	1707 (54.6)

* Numbers represent London boroughs covered by South London and Maudsley (Croydon, Lambeth, Lewisham, Southwark).

† These percentages exclude missing data (Nn=45 845).

‡ These percentages exclude missing data (4.7%) (total Nn=3125).

Table 2 shows the breakdown of ethnicity by type of study (interventional vs observational) among the studies that responded. Missing data rates were 5.5% in observational and 3.7% in interventional studies.

**Table 2:** High-level ethnicity comparison by type of study

Ethnicity	Interventional(n=1494)	Observational(n=1785)
Any Asian or Asian British background	114 (7.6)	79 (4.4)
Any Black or Black British background	328 (22.0)	465 (26.1)
Any Mixed background	113 (7.6)	128 (7.2)
Any other ethnic groups	130 (8.7)	61 (3.4)
Any White or White British background	754 (50.5)	953 (53.4)
Not known	55 (3.7)	99 (5.5)

The 2021 and 2011 Census offered 19 ethnic minority subcategories, in addition to the high-level categories, for respondents to select from, which can be viewed in online supplemental material 3. Data on the Census subcategories are not recorded in the Trust and few of the studies offered participants the option to select from categories similar to the Census subcategories. Ethnic minority subcategories are presented alongside Census data in online supplemental material 5.

In some instances, the number of recruited participants reported by the PI did not match those recorded on the Trust’s EDGE database. Where this happened, we checked such a discrepancy together with the PI; in some cases, this was due to attrition post-recruitment, while in others, PIs only reported on participants recruited directly from the Trust services but did not provide data on those recruited through other means (advertising or through Patient Identification Centre sites, etc). One study provided demographic data on parents, but the recruitment numbers recorded on EDGE were of both parents and children. One study had demographics for higher numbers than recorded on EDGE due to reconsenting participants into a follow-up study; all participants were included as the groups could not be distinguished.

## Discussion

In this survey, we found much higher alignment in relation to ethnicity between participants and Trust population than expected from the limited existing literature.^6 10 16 17^ This is positive in terms of the validity of the evidence generated in the Trust and its applicability to the population we serve. Our findings that 52.1% of participants identify as white are more encouraging than the 94.6% reported in the Pedersen *et al* review^10^ and the 76.5% reported in a recent review of mental health research studies conducted during the perinatal period in the USA and Canada.^18^

The only population where research participation metrics did not align with the Trust patient population are those reporting Mixed heritage, although research participation numbers align well with the wider local population. A possible explanation could be inconsistent categorisation of this group, but this will be important to observe over time.^19^ Further, recorded numbers of research participants from some minorities, including Gypsy, Roma, Traveller and Arab ethnicity, were low, but we do not have Trust data on these ethnic groups, and many studies did not offer these categories, so we are unable to conclude whether this reflected under-representation. Additional data is clearly needed, as populations such as Irish travellers have extremely poor health outcomes.^20^,^22^

That three eligible studies did not collect ethnicity data highlights the longstanding concern of under-recording of ethnicity,^23^ although ample guidance has been published in recent years.^24^,^27^ One problem is that individuals are not routinely asked about ethnicity, and there are no national standards to classify it and distinguish it from race.^24^ Another issue is participants not reporting their ethnicity. Possible explanations include reservations or concerns about how data might be used^28^ and the complexity of ethnic backgrounds, making it difficult for participants to provide a response, particularly if there is no self-reporting option.^29^ Offering an explanation as to why ethnicity is asked can lead to higher response rates.^30^

### Strengths and limitations

This is, to our knowledge, the first study to compare the ethnic representativeness of research participants across a large mental healthcare provider with the clinical population and local population served. The study took place in the UK mental health Trust with the greatest number of studies and participants recruited to research.^31^ Our team had access to comprehensive comparator figures from both the Trust and the local Census data for the catchment area population served.

Along with comprehensive services to four South East London boroughs, the South London and Maudsley NHS Trust also provides addiction services to three other boroughs, Wandsworth, Greenwich and Bexley, along with several National and Specialist services. The population served through these services is less diverse than the local population and thus would not have amplified the diversity of our research participants.

In terms of limitations, six studies did not respond to our query. Additionally, there was varying categorisation of ethnicity across studies, reflecting a general issue associated with ethnicity reporting in clinical research.^16^ For reasons of timing, no eligible studies were on the mental health of older adults, which area will require examination when routinely collected data becomes available at a sufficient scale.

To preserve patient anonymity, this survey was retrospective and limited to ‘large’ closed studies, with numbers relating to groups of less than five removed. What is needed is routine recording of demographic data, not just for race and ethnicity but also for other protected characteristics. EDGE has recently started recording ethnicity data routinely, so representativeness will be easier to assess in future.

## Conclusion

According to our survey**,** participation in mental health research in this large urban mental health Trust appears representative of the diversity of the population served. While this is encouraging, there is a clear need for continuous monitoring. Future efforts should prioritise consistent, respectful and inclusive reporting of participants’ ethnic backgrounds in mental health research to reflect our rich, ethnically diverse communities across healthcare systems. Only by doing this will we be able to identify and, where relevant, address ethnically framed health inequalities.

## Supplementary material

10.1136/bmjopen-2024-093269 online supplemental file 1

## Data Availability

Data are available upon reasonable request.
