# Supplementary material for: Does the ethnicity distribution of research participants reflect the eligible population? Survey of participants recruited through a UK mental health Trust
Source: BMJ Open. 2025 Mar 19;15(3):e093269. doi: 10.1136/bmjopen-2024-093269 (PMC12004483; doi:10.1136/bmjopen-2024-093269)
Supplement: online supplemental file 1 [file bmjopen-15-3-s001.docx]

## **SUPPLEMENTARY MATERIAL**

##

## **Supplementary Material 1: Search strategy**

## The list of studies was derived from the EDGE database. EDGE is a widely used clinical research management system employed widely across the NHS to record recruitment into studies and developed by the Clinical Informatics Research Unit at the University of Southampton (<https://www.hdruk.ac.uk/organisations/edge/>). Due to the database’s interface, searches are done manually using the available filters.

## The filters used were:

## Participant number >/=90 recruited through the South London and Maudsley NHS Foundation Trust

## Study opened on or after 1st April 2012

## Study closed on or before 1st April 2022

## Data extracted (manually) included:

## Name of study

## Name of local Principal Investigator

## Type of study (interventional/ observational and single or multi-centre

## Clinical Academic Group in which study was registered

## Number of recruited participants

## **Supplementary Material 2: Study data groupings**

White included:

- White (not specified)
- White British
- White Irish
- White (Other)
- Mediterranean (e.g. Turkish, Greek, Italian)

Black included:

- Black (not specified)
- Black (African)
- Black (Caribbean)
- Black (Other)

Asian included:

- Asian (not specified)
- Asian/Asian British
- Indian Subcontinent
- Bangladeshi
- Indian
- Pakistani
- Chinese
- Asian (Other)

Mixed Ethnicity included:

- Mixed/Multiple Ethnic Groups
- Mixed/White & Asian
- Mixed/White & Black African
- Mixed/White & Black Caribbean
- Mixed (Other)

Other ethnic groups included:

- Arab
- Middle-Eastern
- Hispanic
- Latino
- Other (not White British)
- Other (not specified)

Not included in categorisation and analysis:

- British (not specified)
- English (not specified)

## **Supplementary Material 3: Census 2021 categories**

Asian or Asian British

- Indian
- Pakistani
- Bangladeshi
- Chinese
- Indian subcontinent
- Any other Asian background

Black, Black British, Caribbean, or African

- Caribbean
- African
- Any other Black, Black British, or Caribbean background

Mixed or multiple ethnic groups

- White and Black Caribbean
- White and Black African
- White and Asian
- Any other Mixed or multiple ethnic background

White

- English, Welsh, Scottish, Northern Irish or British
- Irish
- Gypsy or Irish Traveller
- Roma
- Any other White background

Other ethnic group

- Arab
- Any other ethnic group

## **Supplementary Material 4: Detailed ethnicity breakdown**

**Table 3. Detailed ethnicity breakdown of research participants (N=3279)**

| **Ethnicity** | **Research** |
| --- | --- |
| Asian (not specified) | 73 (2.2%) |
| Asian/Asian British | 13 (0.4%) |
| Bangladeshi | 14 (0.4%) |
| Indian Subcontinent | <5 |
| Indian | 36 (1.1%) |
| Pakistani | 10 (0.3%) |
| Chinese | 9 (0.3%) |
| Asian (other) | 33 (1.0%) |
| Black | 174 (5.3%) |
| Black (African) | 259 (7.9%) |
| Black (Carribbean) | 228 (7.0%) |
| Black (Other) | 132 (4.0%) |
| Mixed/Multiple Ethnic Group | 94 (2.9%) |
| Mixed (White & Asian) | 14 (0.4%) |
| Mixed (White and Black African) | 41 (1.3%) |
| Mixed (White and Black Caribbean) | 36 (1.1%) |
| Mixed (Other) | 56 (1.7%) |
| Hispanic | 5 (0.2%) |
| Latino | <5 |
| Arab | <5 |
| Middle-Eastern | <5 |
| Other (not White British) | 82 (2.5%) |
| Other | 100 (3.0%) |
| White (not specified) | 883 (26.9%) |
| White British | 669 (20.4%) |
| White Irish | 29 (0.9%) |
| White Other | 122 (3.7%) |
| Mediterranean (e.g. Turkish, Greek, Italian) | <5 |
| White (not specified) | 883 (26.9%) |
| Declined/DNA/Missing | 154 (4.7%) |

| Supplementary Material 5  Table 4. Detailed ethnicity in Census population and research participants (missing data excluded) | | |
| --- | --- | --- |
| Ethnicity | **South East London population** | **Research population (N=1880)*** |
| Asian, Asian British or Asian Welsh: Bangladeshi | 13424 (1.0%) | 14 (0.7%) |
| Asian, Asian British or Asian Welsh: Chinese | 23637 (1.8%) | 9 (0.5%) |
| Asian, Asian British or Asian Welsh: Indian | 46808 (3.6%) | 36 (1.9%) |
| Asian, Asian British or Asian Welsh: Pakistani | 23580 (1.8%) | 10 (0.5%) |
| Asian, Asian British or Asian Welsh: Other Asian | 41556 (3.2%) | 33 (1.8%) |
| Black, Black British, Black Welsh, Caribbean or African: African | 163732 (12.4%) | 259 (13.8%) |
| Black, Black British, Black Welsh, Caribbean or African: Caribbean | 115138 (8.8%) | 228 (12.1%) |
| Black, Black British, Black Welsh, Caribbean or African: Other Black | 43488 (3.3%) | 132 (7.0%) |
| Mixed or Multiple ethnic groups: White and Asian | 19293 (1.5%) | 14 (0.7%) |
| Mixed or Multiple ethnic groups: White and Black African | 15802 (1.2%) | 41 (2.2%) |
| Mixed or Multiple ethnic groups: White and Black Caribbean | 33855 (2.6%) | 36 (1.9%) |
| Mixed or Multiple ethnic groups: Other Mixed or Multiple ethnic groups | 32829 (2.5%) | 56 (3.0%) |
| White: English, Welsh, Scottish, Northern Irish or British | 486642 (37.0%) | 669 (35.6%) |
| White: Irish | 22842 (1.7%) | 29 (1.5%) |
| White: Gypsy or Irish Traveller | 630 (0.1%) | <5 |
| White: Roma | 5269 (0.4%) | <5 |
| White: Other White | 161349 (12.3%) | 122 (6.5%) |
| Other ethnic group: Arab | 9702 (0.7%) | <5 |
| Other ethnic group: Any other ethnic group | 56991 (4.3%) | 191 (10.2%) |

*N.B.: Percentages calculated based only on number of participants whose ethnicity was recorded under the 19 Census ethnicity subcategories (N=1880); missing data or high-level ethnicity data have been excluded.
