# Supplementary material for: Does the ethnicity distribution of research participants reflect the eligible population? Survey of participants recruited through a UK mental health Trust
Source: BMJ Open. 2025 Mar 19;15(3):e093269. doi: 10.1136/bmjopen-2024-093269 (PMC12004483; doi:10.1136/bmjopen-2024-093269)
Supplement: Supplementary file 2 [file bmjopen-2024-093269.reviewer_comments.pdf]

PEER REVIEW HISTORY

BMJ Open publishes all reviews undertaken for accepted manuscripts. Reviewers are asked to complete a checklist review form and are provided with free text boxes to elaborate on their assessment. These free text comments are reproduced below.

ARTICLE DETAILS

Title (Provisional)

Does the ethnicity distribution of research participants reflect the eligible population? Survey of participants recruited through a UK mental health Trust

Authors

Dima, Aikaterini; Brown, Amanda; Shlovogt, Tanya; Martinez, Silian; Onwumere, Juliana; Nettis, Maria Antonietta; Chua, Kia-Chong; Hotopf, Matthew; Gaughran, Fiona

---

VERSION 1 - REVIEW

**Reviewer**	**1**
**Name**	**Amano, Takashi**
**Affiliation**	**Rutgers University Newark**
**Date**	**20-Oct-2024**
**COI**	**None**

I would like to thank the authors for their valuable manuscript examining the representation of ethnic minorities in mental health research, using data on participation from studies recorded in the South London and Maudsley NHS Foundation Trust system. I believe this manuscript has two major issues.

1. Scientific significance of the findings: My primary concern is the lack of clarity regarding the significance of this study. The introduction does not clearly explain why it is important to "examine whether research participation reflects the population we serve in terms of ethnicity." The previous studies cited by the authors already document the underrepresentation of ethnic minorities in research. Please provide more background on the aim of this study. Are the authors assessing the effectiveness of the NIHR's "steps to promote inclusive research recruitment"? What are the specific research questions and hypotheses?

2. Implications of the study: The analysis and results presented in this manuscript appear too simplistic to provoke meaningful discussion. What distinguishes this study from previous research, which reported much lower percentages of ethnic minority participation? I am particularly interested in the trends over time in ethnic minority representation. Has it been

increasing or decreasing? Additionally, how do the research topics and geographic locations of the studies influence the findings?

Reviewer 2

Name Fulda , Kimberly G

Affiliation Department of Family Medicine and Osteopathic Manipulative Medicine, The North Texas Primary Care Practice-Based Research Network (NorTex), University of North Texas Health Science Center, Fort Worth, TX, USA.

Date 20-Dec-2024

COI None

Thank you for the opportunity to review the article titled "The Representation of Ethnic Minorities in Mental Health Research: Data from South London and Maudsley NHS Foundation Trust." The article addresses an important topic that is under researched. Inclusion of all racial/ethnic groups is crucial to ensure the research results are generalizable and helps reduce disparities in medical care. Please see the following comments/suggestions.

It would be interesting to have additional information on the research studies included. For example, the authors state that the projects were identified using the Trust's clinical management system. Are all of the studies that were selected related to mental health research, or were some of them related to addiction research or a combination? It would also be interesting to know the age range of the research. Did the authors include studies that only had adult participants, or did the studies include all ages?

The authors state that studies who preferentially recruited from a specific ethnic group were excluded, but did any of the studies use a stratified approach to recruit a set number of participants from different ethnic groups?

How many participants were included from observational versus interventional studies, and what was the ethnicity breakdown between the two study categories?

VERSION 1 - AUTHOR RESPONSE

Reviewer: 1

Comments to the Author:
I would like to thank the authors for their valuable manuscript examining the representation of ethnic minorities in mental health research, using data on participation from studies

recorded in the South London and Maudsley NHS Foundation Trust system. I believe this manuscript has two major issues.

We thank the reviewer for their comments.

1. Scientific significance of the findings: My primary concern is the lack of clarity regarding the significance of this study. The introduction does not clearly explain why it is important to "examine whether research participation reflects the population we serve in terms of ethnicity." The previous studies cited by the authors already document the underrepresentation of ethnic minorities in research. Please provide more background on the aim of this study. Are the authors assessing the effectiveness of the NIHR's "steps to promote inclusive research recruitment"? What are the specific research questions and hypotheses?

We thank the reviewer for raising these important points. We address the significance of the study in our introduction particularly with regards to why representative research participation is essential (lines 87-88, 95-104, 110-113, 115-116) and have added/edited as below for clarification.

"Clinical research informs healthcare practice, access and provision, with the goal of improving outcomes for all health care users. Therefore, it has been recognised that inclusion in research should reflect the relevant population diversity in demographic, social and cultural backgrounds (1-4). Despite this, the limited strategies to increase diversity of recruitment (5) have not yet translated to increased participation of minoritised ethnic groups in clinical research (6-8). This under-representation risks systematic ethnic inequalities in clinical care (3, 9) creating a potential gap between available research findings and their applicability to multi-ethnic populations, especially in relation to personalised clinical care.

This gap has also been reported in mental health research. A 2022 quantification (10) examining the demographics of participants in psychiatric research published in the American Journal of Psychiatry over a span of 2 years, found that people categorised as from Asian, Black and minoritised ethnic groups were underrepresented in research. Across 125 studies included, only 43% presented information on participant ethnicity and in those, 94.6%, were described as White or Caucasian. This figure dropped to 74.1% when only non-genetic studies were examined, with 10.6% described as "other" or "nonwhite", 7.6% as African American, Black, or African and 5.3% as Hispanic or Latino. Such metrics risk diminishing the confidence of people from racially minoritised ethnic groups that the knowledge derived from research is relevant to their health needs. This is especially relevant in light of the 2022 UK National Health Service (NHS) Race & Health Observatory report (13), where barriers and disparities in accessing evidence-based mental health services for people from minoritised groups are highlighted.

Minoritised ethnic groups represent approximately 18% of the population of England and Wales (11) and 24.5% of the USA population (12). While the National Institute for Health and

Care Research (NIHR) (14) in England has taken steps to promote inclusive research recruitment (15), we aimed at establishing historical research representativeness in a large urban NHS Mental Health Trust over a 10-year period.

The main objective of our study was thus to examine whether research participation in a UK mental health setting reflects the population served in terms of ethnicity."

We wish to also clarify that we are not assessing the effectiveness of the NIHR policy but rather examining our own internal research output as one of the largest academic centres for mental health worldwide. It is particularly because of the repeated documented underrepresentation of ethnic minorities in research and the effect this has on patient confidence in our evidence-based practice. Representation becomes even more important in academic institutes whose output might and does inform practice guidelines.

Due to the methodology of this study (survey) and limited statistical analysis, there is no null hypothesis to be rejected. The wider assumption was that the survey would produce similar findings to the other research on ethnic representation, although we were aware that at least some studies had appropriately diverse representation. On completion, we were pleased to find that representation was well matched.

We wish to further note that there is a paucity of studies examining representation specifically in mental health research making our study the first of its kind, as we have noted in lines 272-274.

2. Implications of the study: The analysis and results presented in this manuscript appear too simplistic to provoke meaningful discussion. What distinguishes this study from previous research, which reported much lower percentages of ethnic minority participation? I am particularly interested in the trends over time in ethnic minority representation. Has it been increasing or decreasing? Additionally, how do the research topics and geographic locations of the studies influence the findings?

We thank the reviewer for their thought-provoking points. Indeed, our methodology and analysis are simple. Following consultation with our expert statistician, we decided against statistical analysis due to differing source populations among Trust patients and research participants. This meant that direct comparisons of confidence intervals would not be possible.

What distinguishes our study from other studies, is primarily that this is the first study examining representation of ethnic minorities in direct comparison with the local population (both within our Trust and our local London Boroughs) specifically in mental health research. Prior published work examined published psychiatric research only. It is also set in the largest UK mental health trust with the largest mental health research output, possibly worldwide. But probably the most important factor is our encouraging results, which we hope will give greater confidence to the populations we serve that diversity is reflected in the research evidence generated in the most productive centre for mental health research in the UK.

We have amended our Strengths and limitations to reflect the above more accurately (lines 274-283).

"The study took place in the UK mental health Trust with the greatest number of studies and participants recruited to research (15). Our team had access to comprehensive comparator figures from both the Trust and the local Census data for the catchment area population served."

Comparisons over time or by research topics were outside the scope of our study (pilot survey) and thus not conducted. This is, however, an interesting point that we will explore in future studies. It will be particularly interesting to explore changes as our research data management system will be incorporating mandatory ethnicity reporting.

London is one of the most diverse cities worldwide, as identified by the Census 2021 data (see Table 1 and online). London's diversity was a key factor in designing this study, as our research outcomes should be applicable to all Londoners - and beyond.

Reviewer: 2

Comments to the Author:
Thank you for the opportunity to review the article titled "The Representation of Ethnic Minorities in Mental Health Research: Data from South London and Maudsley NHS Foundation Trust." The article addresses an important topic that is under researched. Inclusion of all racial/ethnic groups is crucial to ensure the research results are generalizable and helps reduce disparities in medical care. Please see the following comments/suggestions.

We thank the reviewer for their recognition of the importance of representation in research.

Comments:

1. **It would be interesting to have additional information on the research studies included. For example, the authors state that the projects were identified using the Trust's clinical management system. Are all of the studies that were selected related to mental health research, or were some of them related to addiction research or a combination? It would also be interesting to know the age range of the research. Did the authors include studies that only had adult participants, or did the studies include all ages?**

We thank the reviewer for this suggestion, which contributes to improve our study. All of the studies were related to mental health. We have added explanation as to the research population (which also addressed the suggestion on age) in lines 182-184 as follows:

"18 studies were in Adult Mental Health, two studies were in Addictions, five in Children and Adolescent Mental Health, and three studies were jointly between CAMHS and Adult Mental Health."

2. **The authors state that studies who preferentially recruited from a specific ethnic group were excluded, but did any of the studies use a stratified approach to recruit a set number of participants from different ethnic groups?**

We thank the reviewer for this suggestion. None of the studies used a stratified approach to recruit set numbers of participants from different ethnic groups and thus we have not made any amendments.

3. **How many participants were included from observational versus interventional studies, and what was the ethnicity breakdown between the two study categories?**

We thank the reviewer for this suggestion, which provides interesting information for the reader. We have as such incorporated changes in lines 205-207 including a new table, outlining the findings by type of study. The amendments (excluding the table) are copied below:

*"**Table 2** shows the breakdown of ethnicity by type of study (interventional vs observational) among the studies that responded. Missing data rates were 5.5% in observational and 3.7% in interventional studies."*

Comments to the Editor

We would like to report one correction to our manuscript. In line 181 on the number of participants across the 28 eligible studies – this was amended from 4115 to **4856**. We had erroneously reported the total number of participants across the 22 studies that responded but have now provided the correct number.

We have reviewed again all other numbers and percentages provided throughout our manuscript and confirmed that they are all correct.

VERSION 2 - REVIEW

Reviewer 2

Name Fulda , Kimberly G

Affiliation Department of Family Medicine and Osteopathic Manipulative Medicine, The North Texas Primary Care Practice-Based Research Network (NorTex), University of North Texas Health Science Center, Fort Worth, TX, USA.

Date 29-Jan-2025

COI

Thank you for addressing the reviewer comments. I have no further suggested revisions.
